# Identification and verification of a BMPs-related gene signature for osteosarcoma prognosis prediction

**DOI:** 10.1186/s12885-023-10660-5

**Published:** 2023-02-22

**Authors:** Long Xie, Jiaxing Zeng, Maolin He

**Affiliations:** 1 Division of Spinal Surgery, The First Affiliated Hospital of Guangxi Medical University, Nanning, Guangxi Zhuang Autonomous Region China; 2 Trauma Department of Orthopaedics, The Affiliated Yuebei People’s Hospital of Shantou University Medical College, Shaoguan, Guangdong Province China; 3 Trauma Department of Orthopedics, The Fourth Affiliated Hospital of Guangxi Medical University, Liuzhou, Guangxi Zhuang Autonomous Region China

**Keywords:** Osteosarcoma, BMPs, Bioinformation, Nomogram, Risk score model, Immune infiltration

## Abstract

**Background:**

This study aimed to get a deeper insight into new osteosarcoma (OS) signature based on bone morphogenetic proteins (BMPs)-related genes and to confirm the prognostic pattern to speculate on the overall survival among OS patients.

**Methods:**

Firstly, pathway analyses using Gene Ontology (GO) and the Kyoto Encyclopedia of Genes and Genomes (KEGG) were managed to search for possible prognostic mechanisms attached to the OS-specific differentially expressed BMPs-related genes (DEBRGs). Secondly, univariate and multivariate Cox analysis was executed to filter the prognostic DEBRGs and establish the polygenic model for risk prediction in OS patients with the least absolute shrinkage and selection operator (LASSO) regression analysis. The receiver operating characteristic (ROC) curve weighed the model’s accuracy. Thirdly, the GEO database (GSE21257) was operated for independent validation. The nomogram was initiated using multivariable Cox regression. Immune infiltration of the OS sample was calculated. Finally, the three discovered hallmark genes’ mRNA and protein expressions were verified.

**Results:**

A total of 46 DEBRGs were found in the OS and control samples, and three prognostic DEBRGs (DLX2, TERT, and EVX1) were screened under the LASSO regression analyses. Multivariate and univariate Cox regression analysis were devised to forge the OS risk model. Both the TARGET training and validation sets indicated that the prognostic biomarker-based risk score model performed well based on ROC curves. In high- and low-risk groups, immune cells, including memory B, activated mast, resting mast, plasma, and activated memory CD4 + T cells, and the immune, stromal, and ESTIMATE scores showed significant differences. The nomogram that predicts survival was established with good performance according to clinical features of OS patients and risk scores. Finally, the expression of three crucial BMP-related genes in OS cell lines was investigated using quantitative real-time polymerase chain reaction (qRT-PCR) and western blotting (WB).

**Conclusion:**

The new BMP-related prognostic signature linked to OS can be a new tool to identify biomarkers to detect the disease early and a potential candidate to better treat OS in the future.

**Supplementary Information:**

The online version contains supplementary material available at 10.1186/s12885-023-10660-5.

## Background

A high degree of osteosarcoma (OS) malignancy is associated with high mortality. Although innovative treatments for OS have been proposed, standard treatments are still limited to traditional surgery and chemotherapy [1]. The clinical outcome of OS patients, especially those with pulmonary metastases, has not been considerably improved [2]. Furthermore, the underlying molecular mechanisms and potential therapeutic targets behind disease progression have not been fully elucidated. Therefore, identifying both prognostic and predictive markers for OS is significant for diagnosing and treating OS precisely.

Bone morphogenetic proteins (BMPs) are a subgroup of the transforming growth factor-beta (TGF-β) family. BMPs participate in various biological processes (BP), including cell proliferation, differentiation, apoptosis, angiogenesis, migration, and extracellular matrix remodeling [3]. BMP expression is lower at non-union sites and absent at extracellular matrix locations, and no differences between atrophic and hypertrophic non-union tissues were observed [4]. The past two decades have witnessed rapid growth in the amount of clinical application of BMPs due to their ability to induce bone regeneration, especially the recombinant human (rh)BMP-2, which was initially authorized by the FDA in 2002 for single-level anterior lumbar interbody fusion [5]. Given the concerns about cancer and other adverse effects, the utilization of BMP-2, especially in off-label applications, requires robust evidence to ascertain the safety and efficacy of rh BMPs through multicenter, randomized, and double-blind clinical trials [6]. The BMP-signaling pathways can be novel therapeutics for treating chronic diseases that affect the elderly, including osteoporosis and cancer, and are one of the most potent research challenges in medicine [7]. Furthermore, BMP signaling is crucial for regulating osteoclast in osteoporosis treatment from the standpoint of bone homeostasis [8]. Similarly, considering the increased burden of the microscopic residual tumor, BMP-2 is not recommended after limb-salvage surgery in individuals with OS [9]. Most investigations have concentrated on determining the relationship between BMPs as protein factors involving the TGF-β/SMAD signaling pathway and the phenotypic changes of OS [10]. In addition, there has been previous research on the association of different subtypes of BMPs expression and prognosis in different subtypes of OS patients. Subtypes of OS can be identified based on stromal differentiation (osteoblastic, fibroblastic, chondroblastic OS) and tumor features [11]. Based on the immunohistochemistry approach study, the expression of bone morphogenetic proteins, such as BMP-7/8, and their receptors in OS are abnormal and have prognostic significance [12]. OS susceptibility and prognoses in our population may be affected by variations in BMP-2, which is a critical gene for bone formation and maintenance [13]. The Notch signaling plays a key role in OS growth induced by BMP-9 [14]. However, the molecular mechanism by which BMP-related genes are involved in OS pathogenesis remains unclear.

A gene expression profile of OS in public databases was examined to identify differentially expressed BMP-related genes (DEBRGs). Further pathway enrichment analysis was conducted, and the related network of protein–protein interactions (PPIs) was built. Subsequently, combining univariate, multivariate cox regression, and least absolute shrinkage and selection operator (LASSO) regression analysis, an OS prediction signature was generated, and three genes (EVX1, TERT, and DLX2) associated with OS prognosis were found. Furthermore, according to the expression of specific genes and the high- and low-risk scores, Kaplan-Meier (KM) survival analyses were conducted on patients. The results were validated in the additional data sets GSE21257. Additionally, the fraction of 22 tumor-infiltrating immune cells (TIICs) was explored by the CIBERSORT method. Eventually, three hub genes were validated in human OS cell lines using quantitative real-time polymerase chain reaction (qRT-PCR) and western blotting (WB). In this study, a DEBRGs-based prognostic model was formulated, and its prediction accuracy was evaluated in OS patients.

## Methods and materials

### Data collection

The TARGET database (https://ocg.cancer.gov/programs/target) was employed to gather the OS transcriptome data and clinical information of 88 OS patients [15]. The GTEx database was used to acquire gene expression data from 396 healthy human musculoskeletal samples [16]. GSE21257, which contains 53 OS samples from the femur in humans, were used as validation sets [17]. Gene expression profiles for GSE21257 were acquired from the GEO database (GPL10295 platform, Illumina human-6 v2.0 expression beadchip). The GeneCards database (https://www.genecards.org/) yielded 2757 BMP-related genes when the term “bone morphogenetic protein” was searched [18]. All of the genes with a relevance score higher than 0.5 are selected based on “Protein Coding”.

### Identification of DEBRGs

The microarray data were evaluated after normalization in R using the Limma package [19]. The batch effect was removed using the R package “sva”. The “DESeq2” R package was applied to single out DEBRG clusters between OS and control samples with∣log_2_Fold change(FC) | >1 and adj.P-value < 0.05 as the thresholds [19]. The volcano plot and the heatmap were constructed using “ggplot2” and “pheatmap” packages, respectively.

### Functional annotation and pathway enrichment analysis

To reveal the functions of DEBRGs, Gene Ontology (GO) annotation [20] and Kyoto Encyclopedia of Genes and Genomes (KEGG) enrichment investigations were carried out with the package “clusterProfiler” [21]. The GO terms fall into three levels: BP, cellular components (CC), and molecular functions (MF). The KEGG pathway enrichment analysis is useful for describing gene function at the genomic and molecular levels and identifying associated genes. Data with an adjusted P-value of 0.05 are subjected to statistical analysis.

Additionally, the PPI network of DEBRGs was launched employing the STRING database [22]. The visualized PPI network of DEBRGs was performed by the Cytoscape plugin-CytoNCA (version 3.7.2) [23]. Cytoscape plugin-CytoNCA [24] was used to screen the TOP10 degree DEBRGs.

### Establishment of a prognostic model

A univariate Cox regression analysis was conducted on the DEBRGs. Genes with P-values less than 0.05 were linked with OS prognosis. Then, the risk model was then constructed using multivariate Cox analysis. An R package GLMNET (https://CRAN.R-project.org/package=glmnet) and the LASSO method for variable selection and shrinkage were used to design a model for predicting prognosis [25]. Before running the main method with the n-fold value of 10, cross-validation was performed with the cv.glmnet routine to find the penalty regularization parameter. Lambda.min was used to finalize the value, which is the minimum mean error across cross-validations given by lambda [26].

### Validation of the prognostic model

Patients were labeled as high or low-risk based on the median risk score. The survival and the receiver operating characteristic (ROC) curve was created with the R packages “survival” and “pROC”, respectively, to judge the prediction performance of the risk signature. An external validation set was conducted to test the risk model. The predictive model’s performance was evaluated by analyzing areas under the ROC curves (*AUC*). Based on prior work, low, medium, and high diagnostic performance was defined as an AUC value of less than 0.7, between 0.7 and 0.9, or more than 0.9, respectively [27]. A multivariate Cox regression analysis was implemented to uncover independent OS patient prognostic markers. Then, the calibration curve was managed to determine the effectiveness of OS survival nomograms for 1, 3, and 5 years.

### Comparison of the clinical features of individuals at high and low risk

First, we researched the disparities in risk scores among OS patients with distinct clinical characteristics. We used CIBERSORT databases [28] to screen the differential immune cells amid high- and low-risk groups. Furthermore, the ESTIMATE score and tumor purity were compared between high and low-risk groups.

### Cell culture and reagents

Human OS cells U-2OS, HOS, Saos-2, and 143B, as well as human osteoblast cells hFOB1.19, were supplied by the American Type Culture Collection (ATCC, USA). Human osteoblast hFOB1.19 cells, human OS HOS, and U-2OS were nurtured at 37 °C in Dulbecco’s modified Eagle’s medium (DMEM) (Gibco, USA) accompanied by 1% penicillin/streptomycin, and 10% fetal bovine serum (FBS, ExCell, China). Saos-2 cells were kept in McCoy’s 5 A medium (Gibco, USA) enriched with 10% FBS and 1% penicillin/streptomycin at 37 °C. Then, 10% FBS (ExCell, China) and RPMI-1640 medium (Gibco, USA) were exploited to culture 143B cells in an incubator humidified with 5% CO_2_.

### qRT-PCR analysis

The transcription level was determined by qRT-PCR. RNA was reverse-transcribed into cDNA using the Go Taq® qPCR Master Mix (A6002) from Promega. The 2−∆∆Ct technique was harnessed to determine relative levels of RNA expression. The response condition is presented as follows: the cycle was performed at 95 °C for 120 s, followed by 45 cycles at 95 °C and 60 °C for 30 s each. The relative expression of DLX2, EVX1, and TERT were detected. All experiments were conducted in triplicate. Supplementary material Table S1 shows that primers are used in the experiments.

### WB analysis

Cells collected from OS and hFOB1.19 were centrifuged and washed in PBS. Proteins were quantified with Bradford assays (Bio-Rad Laboratories) upon lysis of the cells with ice-cold RIPA buffer (Servicebio G2002). The proteins (30 ug) were extracted using a 10% sodium dodecyl sulfate-polyacrylamide gel (SDS-PAGE). The polyvinylidene fluoride (PVDF) membrane (Servicebio G6015-0.45) was then used to transfer the samples. At room temperature, 5% skim milk (Servicebio G5002) was added to block the membrane for 1 h. After overnight incubation at 4 °C, the specific antibody was washed with TBST (1 × TBS with Tween, Servicebio WGT8220) three times. After incubation for 30 min at room temperature, the secondary antibody was added. We washed the membrane with TBST three times after incubation with the secondary antibody. The protein bands were spotted with the aid of an ECL kit (Servicebio G2014). To semiquantify the relative band intensities, ImagePro Plus 6.0 (Media Cybernetics, Inc.) was employed. Normalization was performed using GAPDH as an internal reference gene.

### Statistical analysis

KM analysis and log-rank testing were utilized to weigh the disparities in OS between patients at low and high risk with R software (3.6.3) and GraphPad (8.0), respectively. Additionally, the independent prognostic variables were identified using univariate and multivariate Cox regressions. We evaluated the risk model’s prediction performance using ROC curves. An analysis of significance was conducted with *P* < 0.05.

## Results

### The identification of DEBRGs and exploration of functional enrichment

**Fig. 1:**
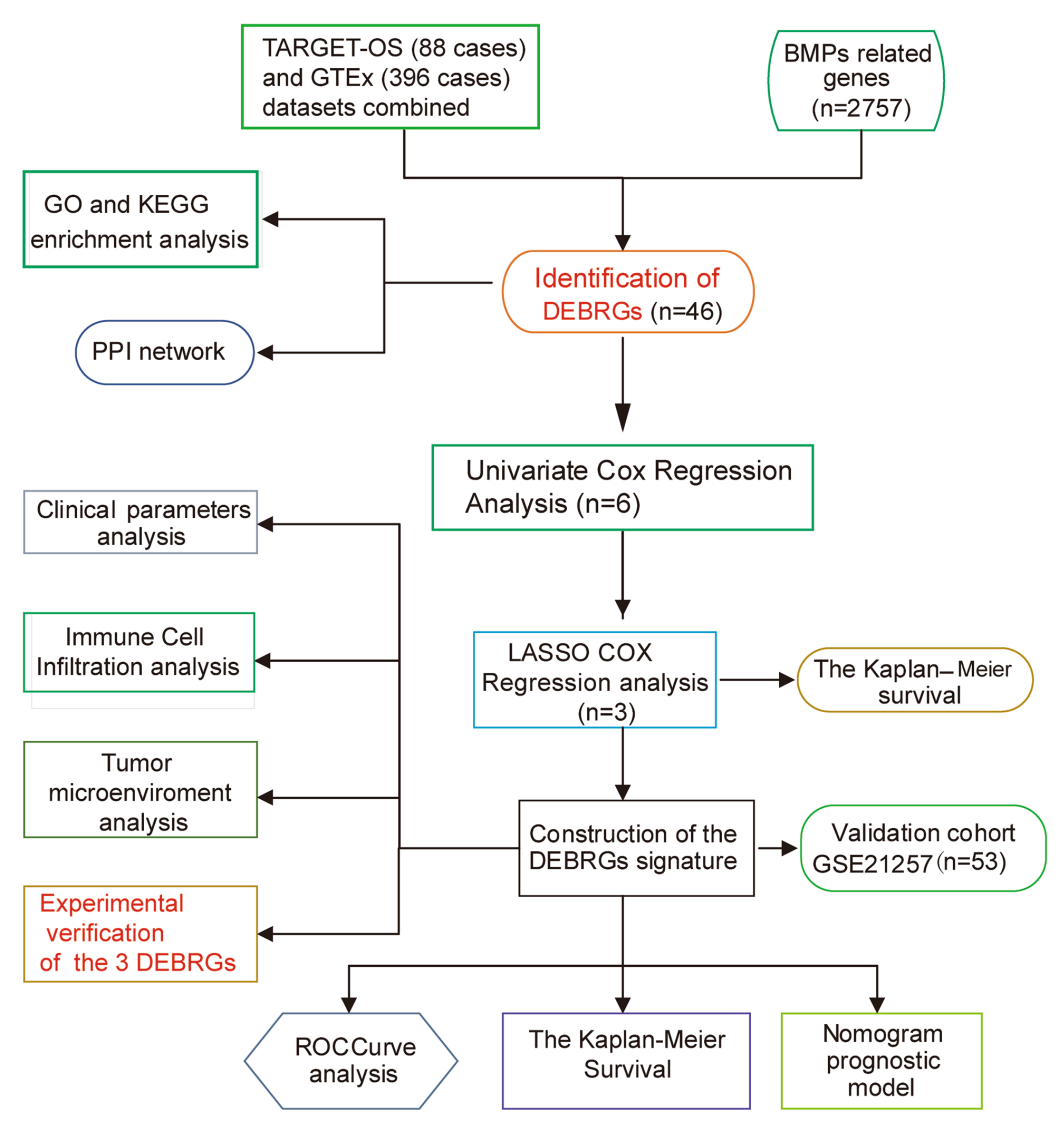
The flow chart of the study AUC = areas under the ROC curves; BMPs = bone morphogenetic proteins; DEBRGs = differentially expressed BMPs-related genes; GO = Gene Ontology; KEGG = Kyoto Encyclopedia of Genes and Genomes; KM = the Kaplan-Meier; LASSO = least absolute shrinkage and selection operator; PPI = protein-protein interaction; WB = western blotting

**Fig. 2:**
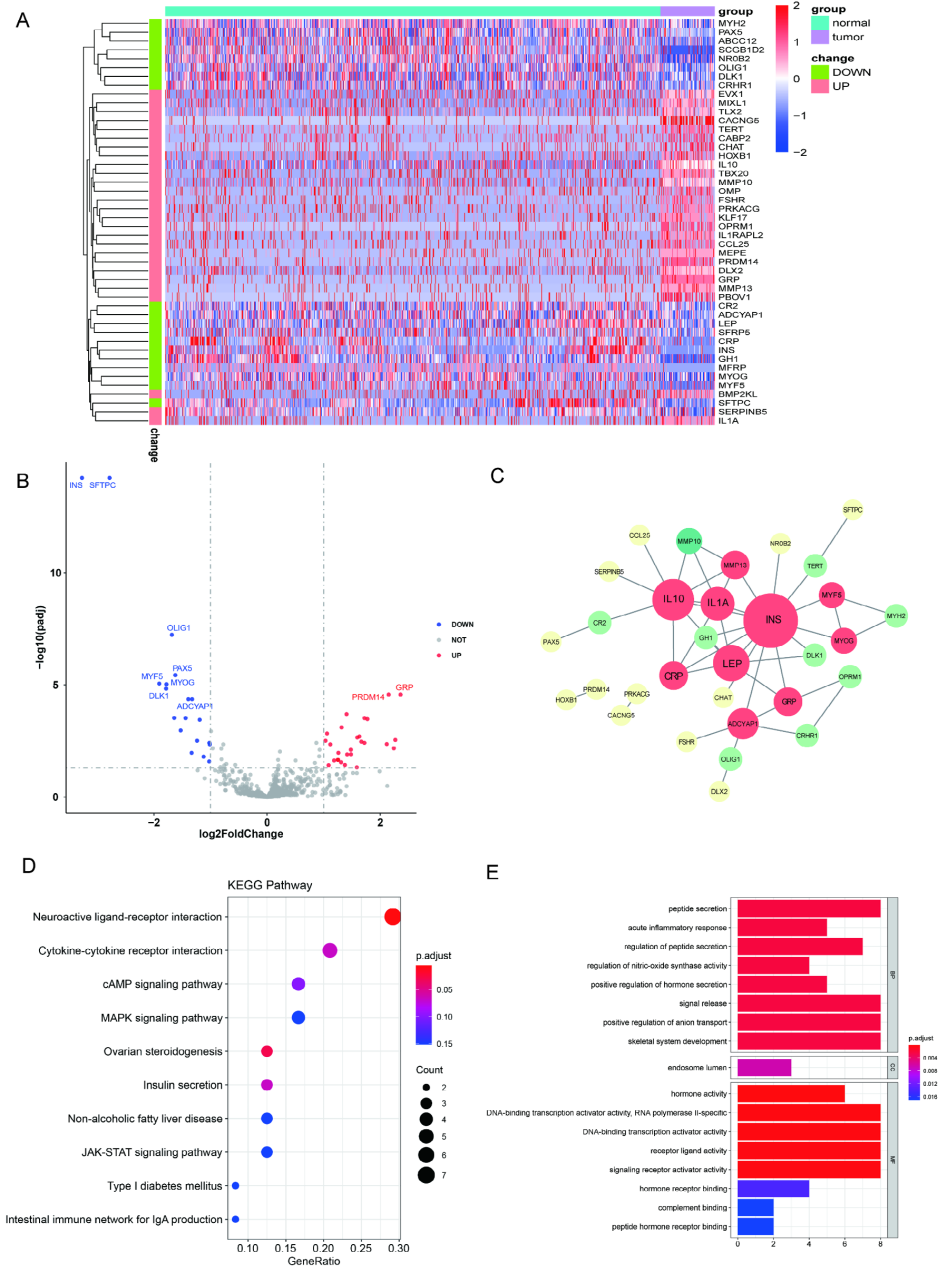
Identification of DEBRGs and functional enrichment analysis. A total of 46 DEBRGs were detected in the **(A)** heat map and **(B)** volcano map (the red dots represent up-regulated DEBRGs, the blue dots represent down-regulated DEBRGs, and the cut-off criteria were set as∣log_2_FC | >1 and adj.*P*. value < 0.05). **(C)** Building PPIs and identifying key modules among the 46 DEBRGs **(D)** KEGG enrichment analysis of DEBRG and **(E)** GO enrichment analysis

This study’s flow chart is shown in Fig. 1. A total of 46 DEBRGs were identified (19 down-regulated genes and 27 up-regulated genes) after the standardization of the microarray results from TARGET-OS. A list of 46 DEBRGs was provided as in Table 1. The heatmap (Fig. 2A) and volcano map (Fig. 2B) display the results.

**Table 1:** 19 down-regulated genes and 27 up-regulated DEBRGs

Gene	log_2_FC	pvalue	padj	change	Gene	log_2_FC	pvalue	padj	change
BMP2KL	1.255526313	0.000488	0.021924	UP	MFRP	-1.64140874	1.74E-06	0.000296	DOWN
PAX5	-1.62253701	6.62E-09	3.67E-06	DOWN	TERT	1.773159318	2.41E-06	0.000333	UP
MMP13	2.266090148	3.07E-05	0.002835	UP	INS	-3.27191889	2.71E-18	5.85E-15	DOWN
MYOG	-1.77926415	2.52E-08	9.31E-06	DOWN	CR2	-1.01372993	6.54E-05	0.004465	DOWN
MEPE	1.48051386	0.000133	0.007755	UP	GH1	-1.23793209	3.58E-05	0.003076	DOWN
MYF5	-1.90650989	1.97E-08	8.72E-06	DOWN	SERPINB5	1.05906509	1.39E-05	0.001469	UP
IL10	1.031964057	3.61E-05	0.003076	UP	GRP	2.357903167	1.07E-07	2.67E-05	UP
DLX2	1.26019405	0.0002	0.010707	UP	PRDM14	2.1488153	1.09E-07	2.67E-05	UP
MIXL1	1.316476512	6.35E-06	0.000782	UP	ABCC12	-1.44081741	2.02E-06	0.000302	DOWN
CHAT	1.375320907	0.00096	0.036662	UP	KLF17	1.720210737	2.05E-06	0.000302	UP
MMP10	1.183810768	0.000552	0.023514	UP	SCGB1D2	-1.02282589	0.000622	0.025994	DOWN
HOXB1	1.404083494	1.08E-06	0.000199	UP	IL1A	1.719261961	4.89E-05	0.003821	UP
LEP	-1.32512567	2.17E-07	4.37E-05	DOWN	OLIG1	-1.68292219	7.86E-11	5.80E-08	DOWN
SFRP5	-1.12169367	0.00033	0.015901	DOWN	CABP2	1.248132368	0.000479	0.021924	UP
DLK1	-1.78484486	4.61E-08	1.46E-05	DOWN	OMP	1.414668811	0.00025	0.012561	UP
CRHR1	-1.01926965	5.18E-05	0.003821	DOWN	MYH2	-1.33368096	0.000203	0.010707	DOWN
CRP	-1.52754551	9.17E-06	0.001068	DOWN	FSHR	1.665802843	4.13E-05	0.003383	UP
TBX20	1.593261403	2.28E-05	0.002199	UP	NR0B2	-1.19161691	2.76E-06	0.000359	DOWN
PRKACG	1.30779911	0.000685	0.028096	UP	OPRM1	1.585800601	0.001342	0.047147	UP
CACNG5	2.239150891	0.000106	0.006727	UP	IL1RAPL2	1.631794914	1.98E-05	0.001992	UP
SFTPC	-2.78453279	5.29E-18	5.85E-15	DOWN	CCL25	1.482475754	0.000241	0.01241	UP
PBOV1	2.115087089	6.09E-05	0.004348	UP	TLX2	1.11780015	6.80E-05	0.004465	UP
EVX1	1.087442919	0.001006	0.037735	UP	ADCYAP1	-1.39311579	2.17E-07	4.37E-05	DOWN

DEBRGs = differentially expressed BMPs-related genes; FC = fold change

A PPI network was cultivated to understand the interplay among the 46 DEBRGs using the STRING online server. Cytoscape plugin CytoNCA was used to screen the TOP10 degree DEBRGs, i.e., INS, IL10, LEP, IL1A, ADCYAP1, MMP13, CRP, GRP, MYOG, and MYF5 (Fig. 2C). Furthermore, the KEGG analysis shows that these genes were important for neuroactive ligand-receptor interaction and ovarian steroidogenesis (Fig. 2D). GO analysis shows that these genes are primarily associated with peptide secretion, endosome lumen, and hormone activity (Fig. 2E). An analysis of the TOP eight KEGG pathway enrichment for DEBRG differential expression is presented in Table 2.

**Table 2:** TOP eight KEGG pathway enrichment for DEBRG differential expression

ID	Description	pvalue	p.adjust	geneID	Count
hsa04080	Neuroactive ligand-receptor interaction	4.12E-05	0.00564	3952/1394/2688/2922/2492/4988/116	7
hsa04060	Cytokine-cytokine receptor interaction	0.001492	0.068138	3586/3952/2688/3552/6370	5
hsa04024	cAMP signaling pathway	0.003618	0.099138	1394/5568/2492/116	4
hsa04010	MAPK signaling pathway	0.010197	0.151619	5568/27,091/3630/3552	4
hsa04913	Ovarian steroidogenesis	0.000434	0.029755	5568/3630/2492	3
hsa04911	Insulin secretion	0.001994	0.068304	5568/3630/116	3
hsa04932	Non-alcoholic fatty liver disease	0.010375	0.151619	3952/3630/3552	3
hsa04630	JAK-STAT signaling pathway	0.011696	0.151619	3586/3952/2688	3

### Construction and verification of a prognostic model

**Fig. 3:**
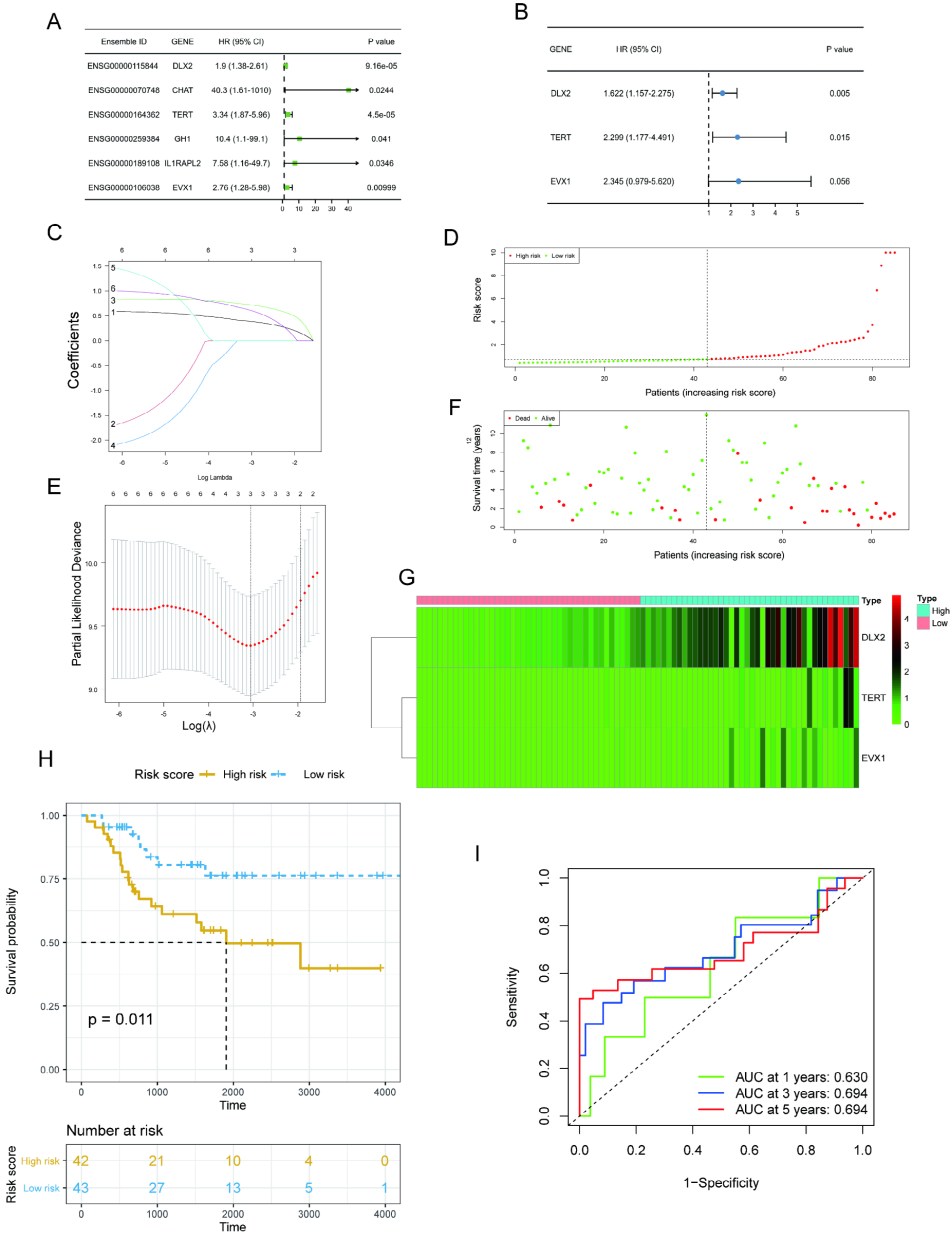
Establishment and validation of the prognostic model. **(A)** Map of the forest for the univariate survival analysis. **(B)** Map of the forest for the multivariate survival analysis. **(C and E)** The construction of a risk model using the LASSO regression analysis. **(D and F)** The number of dead patients increases with the increase of the risk score. **(G)** The risk heat map shows the expression of the three genes from low risk to high risk in the TARGET–OS training set. **(I)** Analyzing ROC curves that predict survival over 1, 3, and 5 years (AUCs of 0.630, 0.694,0.694, respectively) for survival prediction of OS patients on the TARGET–OS training set. **(H)** A survival analysis based on KM methods is conducted on the TARGET–OS training set (*P* < 0.05)

Firstly, a univariate cox regression analysis was executed on the 46 DEBRGs, and six genes with *P* < 0.05 were discovered to be significantly correlated to OS survival (Fig. 3A). The three DEBRGs were applied to generate a risk model (Fig. 3B) after a multivariate Cox regression analysis. Furthermore, the LASSO regression analysis was performed using the R package “glmnet” on the candidate genes identified through the univariate Cox regression analysis. A 10-fold cross-validation was performed to obtain the final regression for three DEBRGs (DEBRGs with higher survival in OS patients) whose coefficients were not penalized. Fig. 3 C and 3E show that DLX2, TERT, and EVX1 are the three DEBRGs. These three gene coefficients seized by the LASSO regression model are shown in Supplement Table 2. In order to identify the risk score, the utilized formula is as follows: The risk score = 0.4837165×DLX2 + 0.8324820×TERT + 0.8523485×EVX1. The samples were divided into high- and low-risk groups according to their median risk score. As shown in Fig. 3G, the three genes are shown from low risk to high risk in the risk heat map. Fig. 3D and 3F show that increasing risk scores are associated with an increase in deaths. Furthermore, the 1-, 3-, and 5-year survival rates were forecasted using the ROC curve analysis. The ROC curve showed that the risk score had a good performance in predicting patients’ survival status. In the TARGET database, the 1-, 3-, and 5-year survival rates were 0.630, 0.694, and 0.694, respectively (Fig. 3I). High- and low-risk individuals differed significantly in their outcomes (*P* < 0.05) using KM analysis (Fig. 3H).

**Fig. 4:**
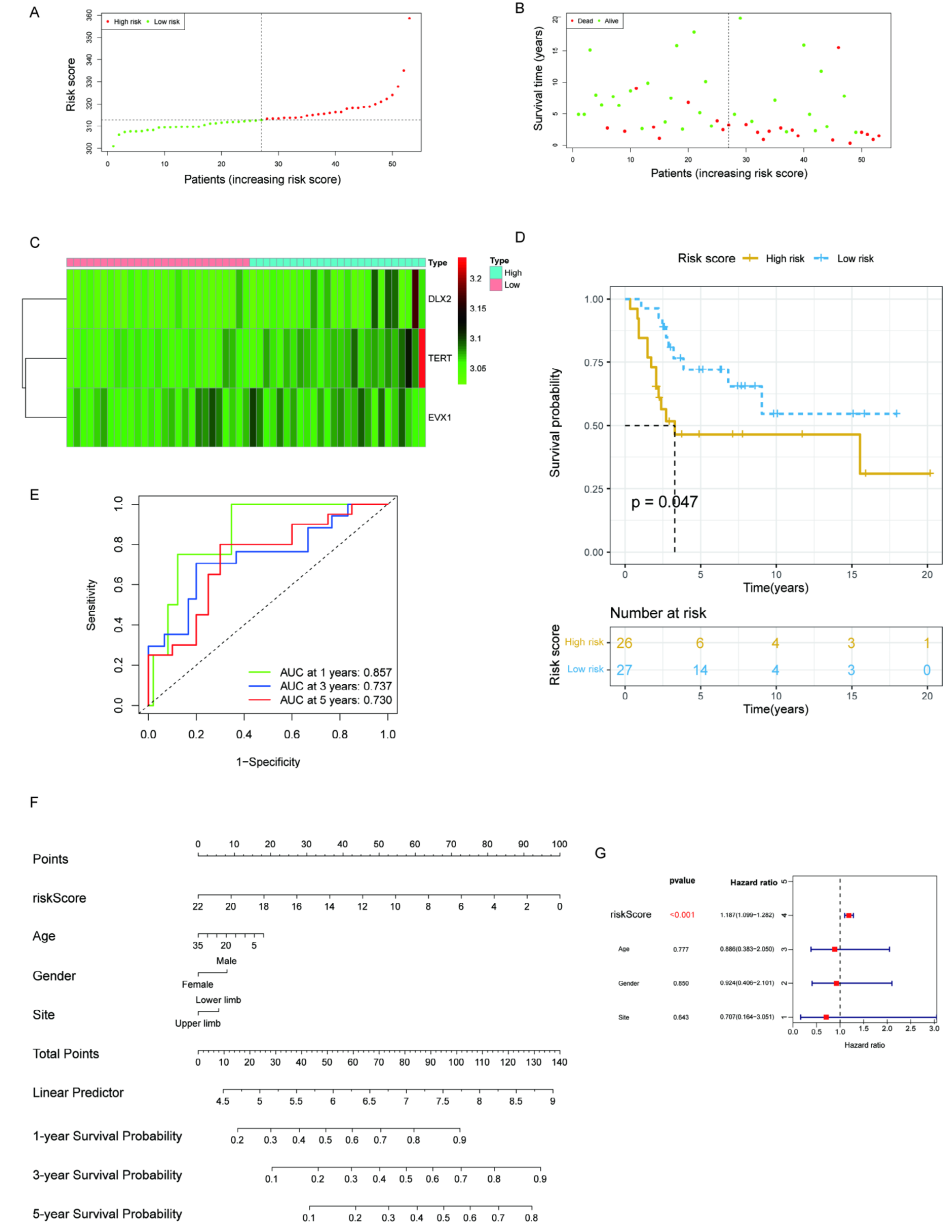
The assessment of the predictive power of the model using the KM analysis and ROC curve in the GSE21257 validation set **(A-B)** The number of dead patients increased with the increase of the risk score. **(C)** The risk heat map shows the expression of the three genes from low risk to high risk in the GSE21257 validation set. **(D)** Survival analysis using KM methods in the GSE21257 validation set (*P* < 0.05). **(E)** An analysis of ROC curves that predict survival rates over 1, 3, and 5 years (AUCs of 0.857,0.737,0.730, respectively) in time-dependent scenarios for survival prediction of OS patients on the GSE21257 validation set. **(F)** Total points are obtained by incorporating the corresponding points of age, gender, site, and risk score on the point scale. **(G)** The multivariate cox regression analysis has identified that risk score is an independent prognostic factor in OS

A median risk score was chosen to classify the GSE21257 dataset into low- and high-risk categories. The number of deaths increased with the increase of the risk score (Fig. 4A-B), and the three genes were expressed in the risk heat map (Fig. 4C) among the two risk groups identified in the validation set of GSE21257, The AUC of the risk score in OS differed significantly between high- and low-risk groups. AUC values were 0.857, 0.737, and 0.730 using ROC curves to forecast the survival rate over 1, 3, and 5 years (Fig. 4E). The KM analysis showed that the overall survival rate differed dramatically between high- and low-risk groups (*P* < 0.05) (Fig. 4D). The risk score is an independent predictor of OS in multivariate Cox regression analysis (Fig. 4G). A nomogram was created to predict OS patient survival over 1, 3, and 5 years using clinical features and risk scores (Fig. 4F).

### Clinical features analysis and immune analysis across the high- and low-risk groups

**Fig. 5:**
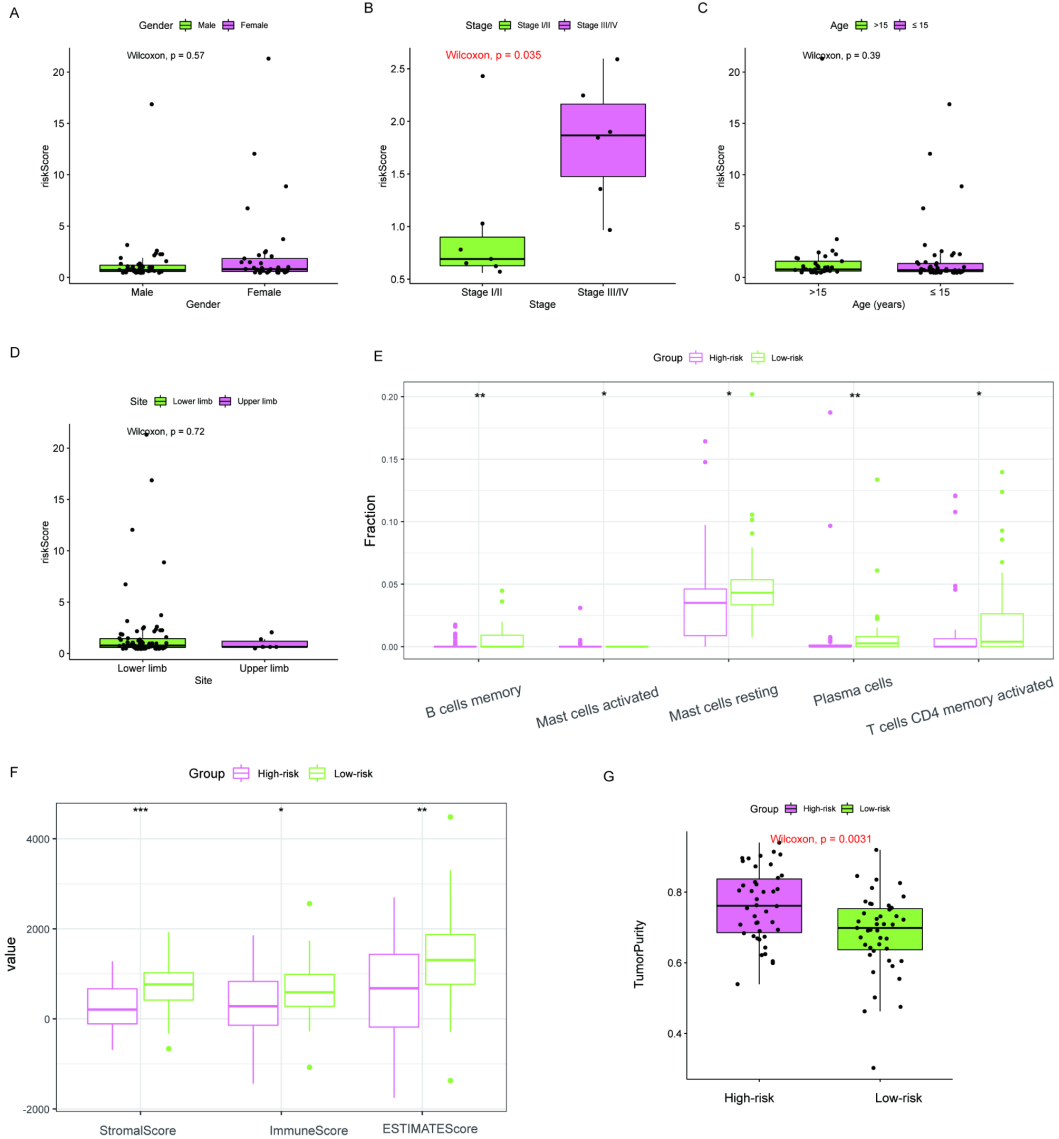
Clinical features analysis and Immune analysis of the high- and low-risk groups. Among all clinical variables: **(A)** gender, **(B)** stage, **(C)** age, and **(D)** site, there is only a significant difference between groups concerning the clinical stage (P < 0.05). **(E)** The infiltration of 22 immune cells and 5 different immune cells (B cells memory, mast cells activated, mast cells resting, plasma cells, and T cells CD4 memory activated) is statistically significant between the high- and low-risk groups. **(F)** The immune scores, stromal scores, and ESTIMATE scores in the low-risk group are significantly higher than those in the high-risk group. **(G)** Compared with the low-risk group, the high-risk group has higher tumor purity (Statistical significance is indicated by ns, no significance; *P < 0.05; **P < 0.01; ***P < 0.001)

In order to investigate the association between a tumor’s prognostic signature and its clinical characteristics, various clinicopathological stratifications were analyzed to determine the distribution of risk scores. The results demonstrated that patients with higher stages had higher risk scores. A statistically significant difference was found between stages I/II and stage III/IV (*P* < 0.05) (Fig. 5A-D). A comparison was made between the infiltration of 22 immune cells in high- and low-risk groups using CIBERSORT. Five different immune cells (memory B, activated mast, resting mast, plasma, and activated memory CD4 + T cells) were obtained (Fig. 5E). The results showed that the low-risk group scored higher on immune, stromal, and ESTIMATE tests (Fig. 5F) and had lower tumor purity than the high-risk group (*P* < 0.05) (Fig. 5G).

### Representative qRT-PCR and WB of expression of three hub signature genes in OS cells

**Fig. 6:**
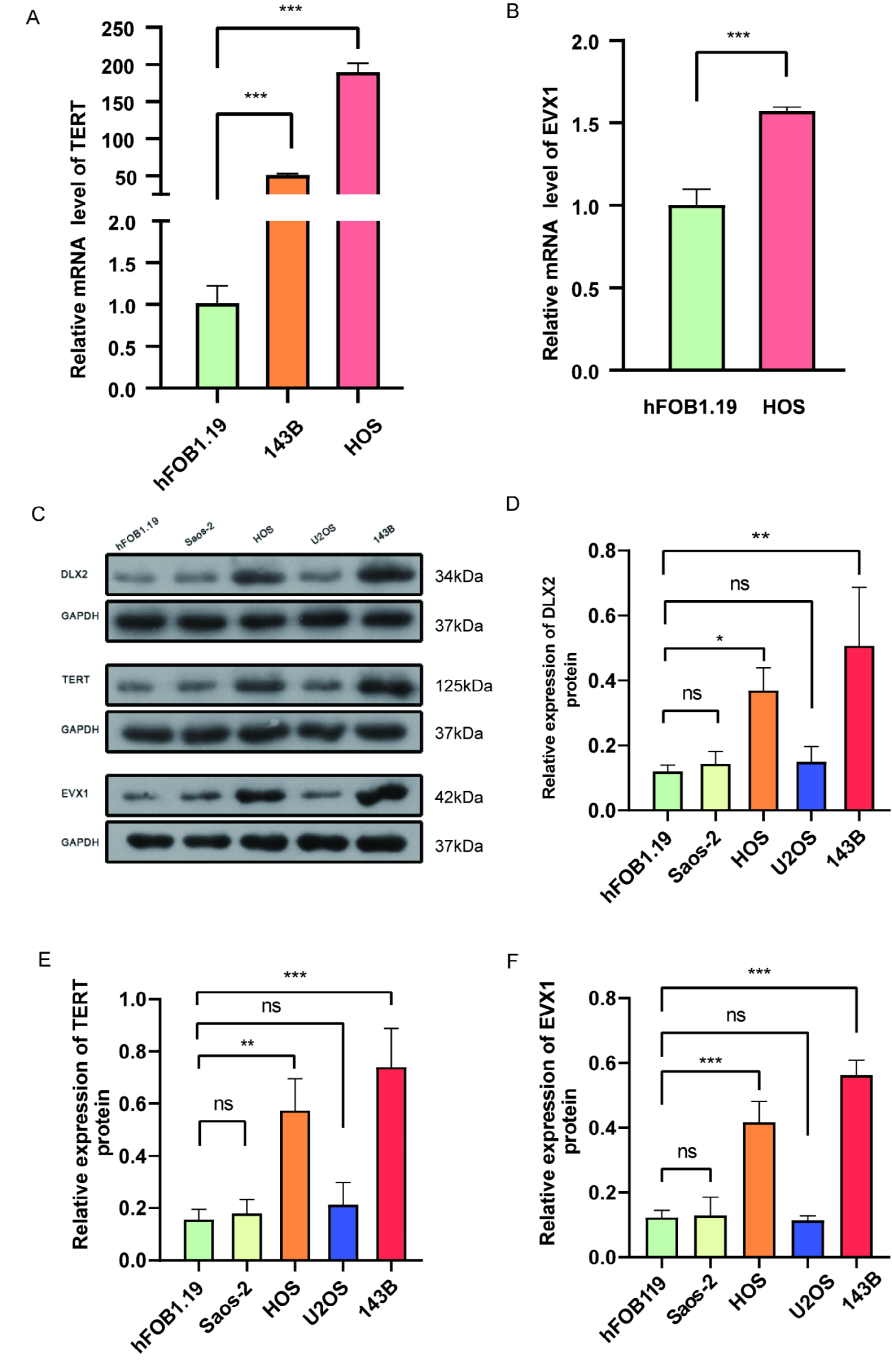
Validation of the critical DEBRGs in the signature. **(A-B)** qRT-PCR is performed to quantify the expression of TERT and EVX1 genes in OS cell lines (143B and HOS) and normal osteoblast cell lines (hFOB1.19). **(C-F)** Western blot is performed to quantify the expression of TERT, EVX1, and DLX2 genes in OS cell lines (143b and HOS), and GAPDH is utilized as the internal control (statistical significance is indicated by ns, no significance; *P < 0.05; **P < 0.01; ***P < 0.001)

A qRT-PCR was performed to investigate the RNA expression levels of EVX1, TERT, and DLX2 genes in human OS cells (143B and HOS) and normal human osteoblasts (hFOB1.19). β-actin was taken as the control. The results showed that TERT was up-regulated in the OS cells (143B and HOS), and the EVX1 gene expression in the OS HOS cell line was higher than that in the hFOB1.19 cells (Fig. 6A-B). A WB analysis was conducted to measure the levels of protein expression of EVX1, DLX2, and TERT in human OS cells. We quantified the protein blots with the ImageJ software and discovered that the expression levels of EVX1, DLX2, and TERT in 143B and HOS OS cells were higher than those in the hFOB1.19 cell line (Fig. 6C-F). These data indicated that EVX1, DLX2, and TERT were up-regulated in human OS cells compared with normal cells (hFOB1.19) (no statistical significance is indicated by ns; *P < 0.05; **P < 0.01; ***P < 0.001). Each experiment was repeated three times.

## Discussion

The OS is one of the highly aggressive cancers characterized by rapid tumor development, recurrence, and metastasis. It is estimated that less than 20% of patients with recurrences or distant metastases will survive 5 years [29]. In the bone microenvironment (BME), BMP2 derived from related cell types functions as potential OS initiating cells to acquire the OS dysregulated phenotypes rather than reduce their tumorigenic potential [30]. Similarly, Gremlin-1 (GREM1), a member of the family of BMP antagonists, is down-regulated in OS cells [31]. The overexpression of GREM1 will impair OS cells’ proliferation, invasiveness, and angiogenesis abilities in vitro and in vivo [32]. Previous studies [33, 34] mainly focused on the BMP-SMAD signaling pathway as a receptor, which was crucial for target gene regulation. Few studies have investigated the association of BMP-related genes in the genesis of OS.

A better understanding of molecular processes underpinning OS occurrence and development has been achieved using bioinformatics analyses [35], which is an emerging field to detect potential diagnostic biomarkers. However, a high false-positive rate and small sample size have been observed when analyzing a single dataset. Therefore, it is necessary to pinpoint novel biomarkers with high specificity, sensitivity, and efficiency for OS diagnosis and prognosis prediction. In this study, DEBRGs were identified, a prognostic model was developed to predict OS patients’ outcomes, and the model was further validated using the GSE21257 dataset.

A total of 46 DEBRGs were identified based on multiple data sets obtained from the GTEx, TARGET, and GeneCards databases. Among the DEBRGs, 27 (58%) were up-regulated and 19 (42%) were down-regulated. Furthermore, we further built a novel risk score model consisting of three DEBRGs calculated by LASSO regression to enhance the predictive performance of the prognostic signature on independent data. Overall survival was better among low-risk patients in the independent test and validation dataset (*P* < 0.05) than that in the high-risk group. Further studies are being conducted on the complex tumor microenvironment (TME), and immunotherapy and tumor modulation may have a clinically significant benefit to OS [36]. Compared with low-risk groups, infiltration of tumor immune cells, including memory B, activated mast, resting mast, plasma, and activated memory CD4 + T cells in high-risk groups was significantly different. These findings are partially consistent with and enrich previous studies [37]. The prognostic risk score model can accurately predict patient survival.

In the present study, the prognostic model consists of three DEBRGs (TERT, EVX1, and DLX2). The three DEBRGs are linked to tumor genesis, progression, and prognosis. The TERT (Telomerase Reverse Transcriptase), a protein-coding gene, can encode the catalytic subunit of telomerase. TERT expression is modulated in tumors by various epigenetic and genetic modifications, and it can influence telomerase activity [38]. Telomerase activity is crucial for most human cancers, mainly due to mutations in its promoter [39]. In OS cells, the silencing of human TERT (hTERT) by shRNA decreases proliferation and increases apoptosis. However, there is a lack of animal studies on hTERT deficiency, and the underlying biological mechanisms remain unclear [40]. EVX1 (Even-Skipped Homeobox 1) can encode a member of the even-skipped homeobox family and is predominantly found in the nucleus and possesses DNA-binding transcription factor activity [41]. Homeobox genes can encode a subset of transcription factors of embryonic development to regulate axial regional specification and can be conveyed erroneously in various solid tumors [42]. Changes in the mRNA expression of EVX1 are associated with clinicopathological features in esophageal squamous cell carcinoma (ESCC). However, the pathogenesis of lymphatic metastasis and tumor invasion still needs further study [43]. In this paper, we have validated the expression of EVX1 in OS based on bioinformatics and cell biology. DLX2 (Distal-less Homeobox 2) can encode a transcription factor that belongs to a member of the Distal-less homeobox transcription factor family. It acts either as an oncogene under abnormal regulation [44] or as a transcription factor that may be engaged in the TGF-β signaling pathway [45].

This is the first study to examine the predictive signature in OS based on DEBRGs. This study, however, still has several limitations. Foremost, our training and validation cohort sample sizes are insufficient to assess the prognostic model’s prediction accuracy fully. Furthermore, the stratification analysis and interaction between the risk factors (for example, clinical prognostic factors and tumor stage) cannot be well represented in the prognostic model. Therefore, more studies with a large sample size are needed to obtain the statistical power to achieve clinically predictive power in clinical applications. How the three hub genes affect OS progression requires experimental validation at molecular, cellular, and organismal levels. Our risk-prediction approach will take longer to implement in the clinic.

## Conclusion

A novel BMPs-associated gene risk signature based on three hub genes (TERT, EVX1, and DLX2) was built based on a combination of bioinformatics analyses. The training and validation sets produced accurate predictions. The results indicate that the model performs well in prognostic studies. Our discoveries can have significant ramifications for the development of new molecular targets for OS immunotherapy and elucidate the clinical prognostic consequences of OS patients in further detail.

## Electronic supplementary material

Below is the link to the electronic supplementary material.

Supplementary Material 1

Supplementary Material 2

Supplementary Material 3

## Data Availability

The datasets generated from The TARGET database https://ocg.cancer.gov/programs/target) and GEO database (https://www.ncbi.nlm.nih.gov/geo/) during and/or analyzed during the current study are publicly available. All raw datas including gels/blots can be obtained from https://www.jianguoyun.com/p/DWHuGG8Ql-GaCxjRlOsEIAA.
