# Supplementary material for: Identification and verification of a BMPs-related gene signature for osteosarcoma prognosis prediction
Source: BMC Cancer. 2023 Feb 22;23:181. doi: 10.1186/s12885-023-10660-5 (PMC9945650; doi:10.1186/s12885-023-10660-5)
Supplement: Supplementary file 1 — Supplementary Material 1 [file 12885_2023_10660_MOESM1_ESM.docx]

**Table S1 LASSO regression model coefficients**

**GENE Coeffocient**

DLX2 0.4837165

TERT 0.8324820

EVX1 0.8523485

**T****able S1** The three gene coefficients obtained by Lasso regression analysis
