# Supplementary material for: Identification and verification of a BMPs-related gene signature for osteosarcoma prognosis prediction
Source: BMC Cancer. 2023 Feb 22;23:181. doi: 10.1186/s12885-023-10660-5 (PMC9945650; doi:10.1186/s12885-023-10660-5)
Supplement: Supplementary file 2 — Supplementary Material 2 [file 12885_2023_10660_MOESM2_ESM.docx]

| **Table S2** The sequences of primers used in this study. | |
| --- | --- |
| β-actin-F | CTCCATCCTGGCCTCGCTGT |
| β-actin-R | ACTAAGTCATAGTCCGCCTAGA |
| DLX2-F | AGCCCCCATCCCTTATCTTA |
| DLX2-R | ATCCGCAAAGGCACCTAAAC |
| EVX1-F | GCAAGTGATGTGTGCGAGGA |
| EVX1-R | TTACCGCCAGATACCGAGAG |
| TERT-F | CACCTGCCGTCTTCACTTCC |
| TERT-R | GTGAACAATGGCGAATCTGG |
