# Supplementary figures and images for: Identification and verification of a BMPs-related gene signature for osteosarcoma prognosis prediction
Source: BMC Cancer. 2023 Feb 22;23:181. doi: 10.1186/s12885-023-10660-5 (PMC9945650; doi:10.1186/s12885-023-10660-5)

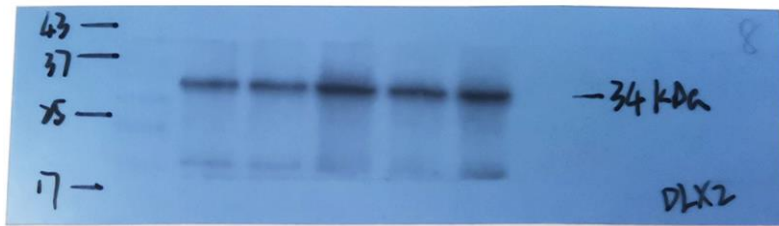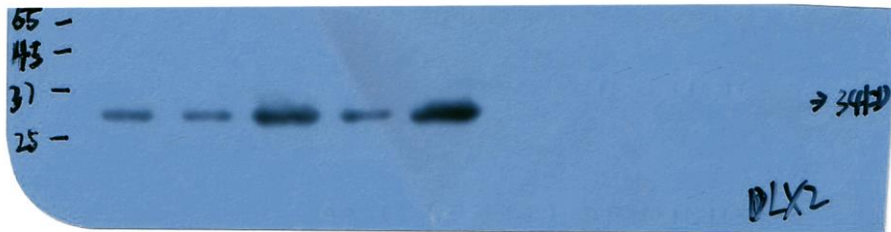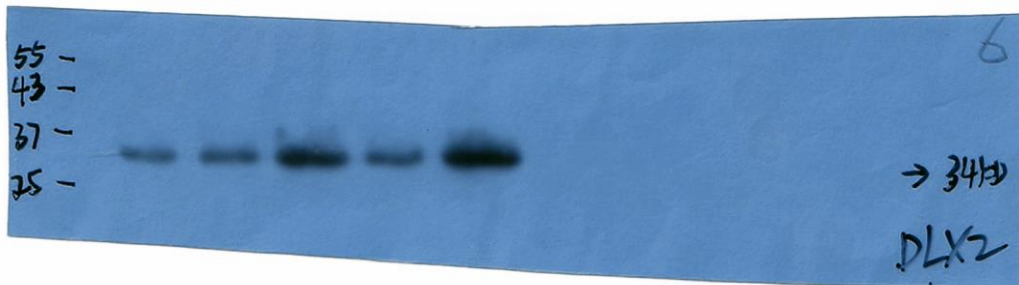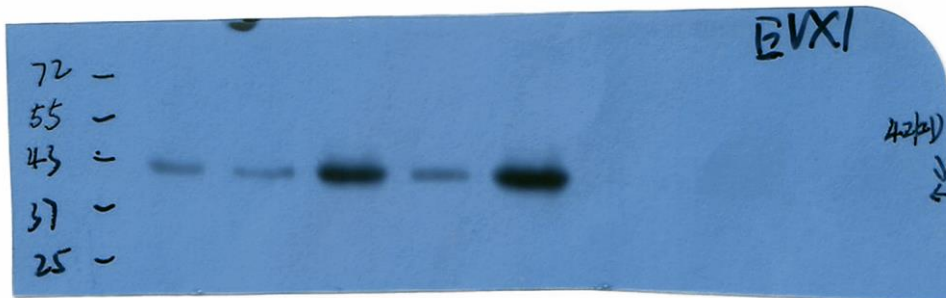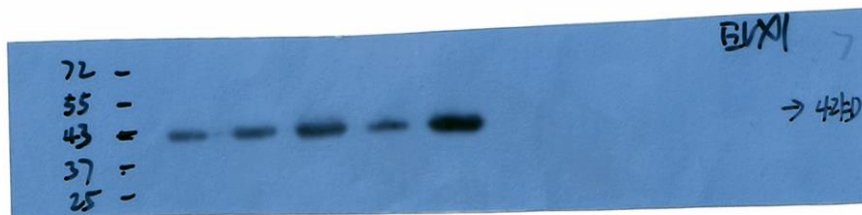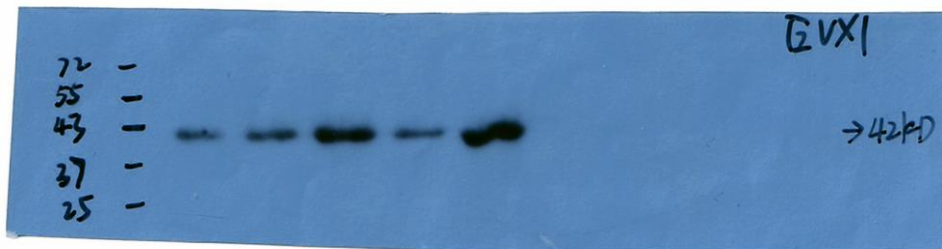

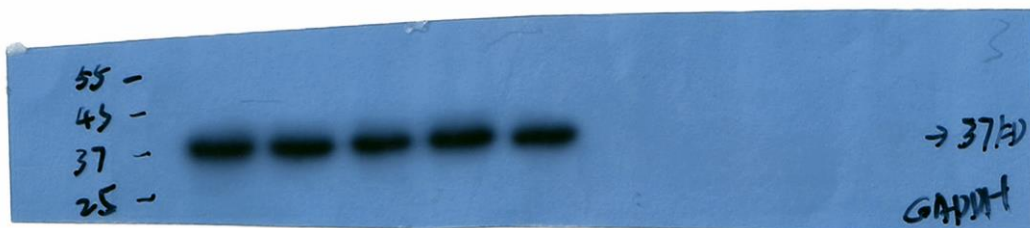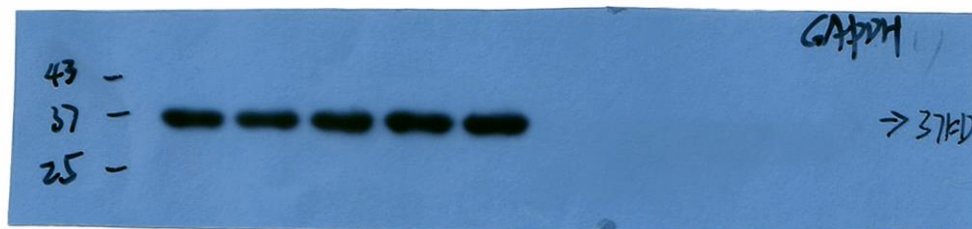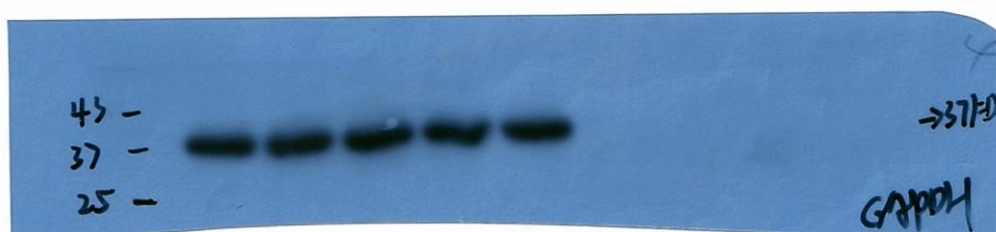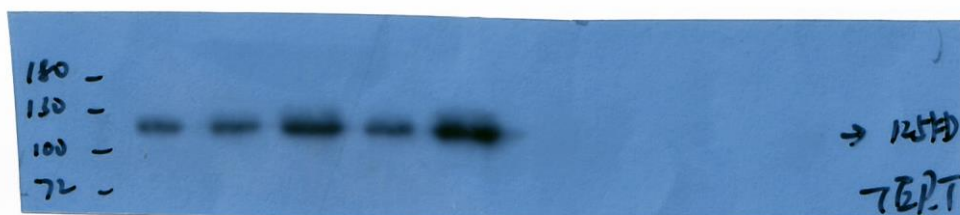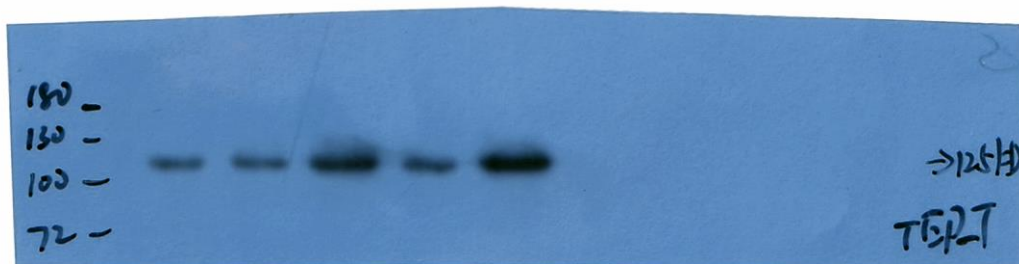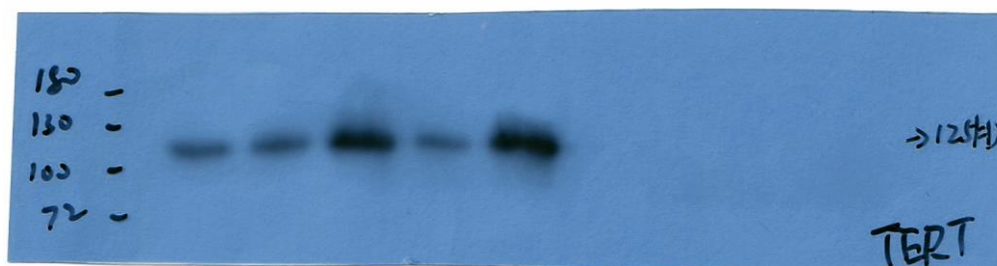

Supplement: Supplementary file 3 — Supplementary Material 3 [file 12885_2023_10660_MOESM3_ESM.pdf]
